# RCSB Protein Data Bank: supporting research and education worldwide through explorations of experimentally determined and computationally predicted atomic level 3D biostructures

**DOI:** 10.1107/S2052252524002604

**Published:** 2024-04-10

**Authors:** Stephen K. Burley, Dennis W. Piehl, Brinda Vallat, Christine Zardecki

**Affiliations:** a Research Collaboratory for Structural Bioinformatics Protein Data Bank, Institute for Quantitative Biomedicine, Rutgers, The State University of New Jersey, Piscataway, NJ 08854, USA; b Research Collaboratory for Structural Biology Protein Data Bank, San Diego Supercomputer Center, University of California, San Diego, La Jolla, CA 92093, USA; c Department of Chemistry and Chemical Biology, Rutgers, The State University of New Jersey, Piscataway, NJ 08854, USA; d Rutgers Cancer Institute of New Jersey, New Brunswick, NJ 08901, USA; University of Auckland, New Zealand

**Keywords:** RCSB Protein Data Bank, experimental structural biology, computed structure models, integrative structures, training, validation protocols

## Abstract

The Protein Data Bank (PDB) was established as the first open-access digital data resource in biology and medicine in 1971 with seven X-ray crystal structures of proteins. Today, the PDB houses >210 000 experimentally determined, atomic level, 3D structures of proteins and nucleic acids as well as their complexes with one another and small molecules (*e.g.* approved drugs, enzyme cofactors). These data provide insights into fundamental biology, biomedicine, bioenergy and biotechnology. They proved particularly important for understanding the SARS-CoV-2 global pandemic. The US-funded Research Collaboratory for Structural Bioinformatics Protein Data Bank (RCSB PDB) and other members of the Worldwide Protein Data Bank (wwPDB) partnership jointly manage the PDB archive and support >60 000 ‘data depositors’ (structural biologists) around the world. wwPDB ensures the quality and integrity of the data in the ever-expanding PDB archive and supports global open access without limitations on data usage. The RCSB PDB research-focused web portal at https://www.rcsb.org/ (RCSB.org) supports millions of users worldwide, representing a broad range of expertise and interests. In addition to retrieving 3D structure data, PDB ‘data consumers’ access comparative data and external annotations, such as information about disease-causing point mutations and genetic variations. RCSB.org also provides access to >1 000 000 computed structure models (CSMs) generated using artificial intelligence/machine-learning methods. To avoid doubt, the provenance and reliability of experimentally determined PDB structures and CSMs are identified. Related training materials are available to support users in their RCSB.org explorations.

## Abstract 2

The RCSB PDB research-focused web portal at https://www.rcsb.org/ provides important tools and resources to search, visualize and analyze experimentally determined 3D biostructures alongside computed structure models of proteins predicted using artificial intelligence/machine-learning based tools.

## 1. Protein Data Bank, wwPDB and the RCSB PDB

### 1.1. PDB has global reach

The PDB was founded in 1971 at Brookhaven National Laboratory on the bedrock values of open access and facile reuse of data (Protein Data Bank, 1971 ▸). The archive is an exemplar of the FAIR [Findability, Accessibility, Interoperability and Reusability (Wilkinson *et al.*, 2016 ▸)] and FACT [FAIRness, Accuracy, Confidentiality and Transparency (van der Aalst *et al.*, 2017 ▸)] principles emblematic of responsible data stewardship in the modern era. Founded with only seven protein structures, PDB holdings have grown over 30 000-fold to more than 210 000 experimentally determined, rigorously validated and expertly curated structures of biological macromolecules.

The PDB has global reach. More than 60 000 structural biologists (‘depositors’) working on every inhabited continent have contributed data to the archive since its inception. This information is used worldwide by many millions of PDB ‘data consumers’ (basic and applied researchers, trainees, educators, and students). The archive has been designated by the Global Biodata Coalition as a Global Core Biodata Resource (https://globalbiodata.org/) and is CoreTrustSeal-certified (https://www.coretrustseal.org/).

PDB data are a public good, which inspired the formation of the Worldwide Protein Data Bank (wwPDB, https://www.wwpdb.org/) organization in 2003 to collaboratively manage the archive (Berman *et al.*, 2003 ▸; wwPDB consortium, 2019 ▸; Velankar *et al.*, 2021 ▸). wwPDB data centers for the PDB Core Archive are responsible for biocuration of regional structure depositions: RCSB PDB [https://RCSB.org/ (Berman *et al.*, 2000 ▸)] for the Americas and Oceania (∼42% of global depositions in 2022); Protein Data Bank in Europe [PDBe, https://pdbe.org (Armstrong *et al.*, 2020 ▸)] for Europe and Africa (∼29% in 2022) and Protein Data Bank Japan [PDBj, https://pdbj.org/ (Kinjo *et al.*, 2017 ▸)] for Asia and the Middle East (∼29% in 2022). Protein Data Bank China (PDBc) was recently admitted as a wwPDB Associate Member (Xu *et al.*, 2023 ▸) and will ultimately assume responsibility for all depositions coming from the People’s Republic of China (∼19% in 2022).

### 1.2. wwPDB core archives

Today, the wwPDB supports three core archives that provide structure data at no charge and with no usage limitations. The PDB stores all atomic coordinates [from macromolecular crystallography (MX), nuclear magnetic resonance spectroscopy (NMR) and 3D electron microscopy (3DEM)] and related experimental data (MX: structure factors, unmerged intensities; NMR: chemical shifts, geometric restraints). The Electron Microscopy Data Bank [EMDB (wwPDB Consortium, 2023 ▸)] stores 3DEM density maps. The Biological Magnetic Resonance Bank [BMRB (Hoch *et al.*, 2023 ▸)] stores spectral and quantitative data derived from NMR studies of biological macromolecules and smaller biomolecules, such as metabolites.

### 1.3. RCSB PDB

RCSB PDB focuses on deposition–validation–biocuration, archive management, enabling public exploration of PDB data, user training and cyberinfrastructure. Our biocuration team works within the wwPDB to the ensure PDB data quality through careful review, format standardization and remediation, and adding metadata annotations that benefit PDB users (Young *et al.*, 2018 ▸).

Over the past years, wwPDB has established domain-specific task forces of subject matter experts to provide recommendations for validating atomic coordinates and the experimental data (Read *et al.*, 2011 ▸; Montelione *et al.*, 2013 ▸; Henderson *et al.*, 2012 ▸). Based on these recommendations, validation protocols have been developed to assess the quality of structures in the PDB (Gore *et al.*, 2017 ▸). In addition to evaluating standard chemical geometry and steric clashes, these protocols include calculation of method-specific refinement statistics and validation metrics. Validation reports are created and made publicly available in the PDB archive to enable consistent evaluation and comparison of structures.

As wwPDB-designated ‘archive keeper’ for the PDB core archive, RCSB PDB is also responsible for PDB data security and updates, releasing >300 new structures biocurated by the wwPDB every Wednesday at 00:00 Universal Time Coordinated.

**Figure 1:**
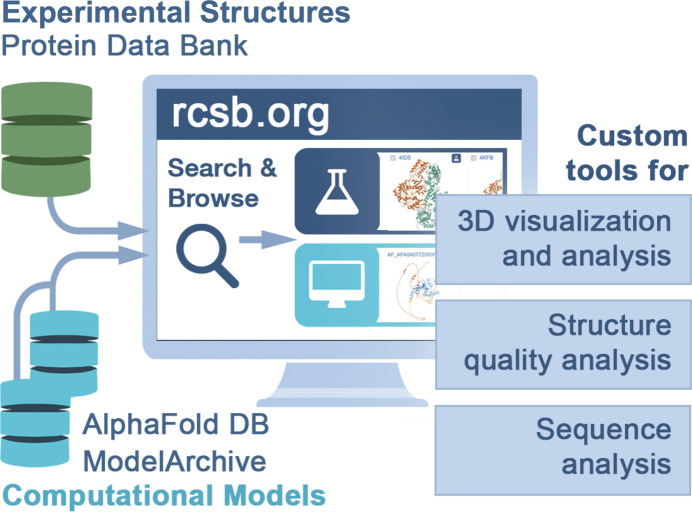
RCSB.org delivers PDB structures (identified with an Erlenmeyer flask icon in dark blue) and CSMs (computer screen icon in cyan) that can be searched, analyzed, visualized and explored using custom tools and features. Image taken from Burley *et al.* (2023 ▸). Published by Oxford University Press on behalf of *Nucleic Acids Research*.

RCSB PDB develops and maintains a research-focused web portal (https://www.rcsb.org/, herein RCSB.org) that supports many millions of users worldwide, representing a broad range of expertise and interests (Burley *et al.*, 2023 ▸). In addition to delivering PDB data, RCSB.org offers comparative data and external annotations, such as information about point mutations and genetic variations, and tools for 2D and 3D visual­ization (Segura *et al.*, 2022 ▸; Burley *et al.*, 2022 ▸; Sehnal *et al.*, 2021 ▸). Alongside PDB structures, the website also provides access to computed structure models (CSMs) generated using artificial intelligence/machine-learning methods (Fig. 1 ▸, see Section 2.1). Value-added comparative data and annotations for both experimental PDB structures and CSMs are updated weekly, ensuring that RCSB.org serves as a living data resource. To further support RCSB PDB users, training and outreach materials are hosted at https://pdb101.rcsb.org/ to help users learn how to utilize PDB data and tell structural biology stories (Zardecki *et al.*, 2022 ▸) (see Section 3.2).

### 1.4. PDB impact: two epidemics to the global pandemic to mRNA vaccines and Paxlovid

The first COVID-19 coronavirus structure was released in record time on 5 February 2020, less than one month after the viral genome sequence became public (PDB entry 6lu7; Jin *et al.*, 2020 ▸). To enable rapid public access to related structures being studied around the world, wwPDB biocurators developed processes and procedures that ensure rapid processing and public release of pandemic-related 3D biostructure data.

RCSB PDB also mounted campaigns to help fight the pandemic. Effective RCSB PDB tools for searching, analyzing and visualizing structures were already in place to help researchers understand coronavirus protein structure–function relationships, design new vaccines, identify potential drug discovery targets, and support drug repurposing and structure-guided discovery of new anti-viral agents. Original PDB-101 content relating to SARS-CoV-2 was developed to support the general public in their sudden crash course in structural biology [*e.g.* measures to interdict viral transmission, structure-guided drug discovery focused on essential viral enzymes and coronavirus biology more broadly (Zardecki *et al.*, 2022 ▸; Goodsell *et al.*, 2020 ▸ *a*)]. A 30% increase in website traffic was recorded in 2020 (versus 2019). RCSB PDB student mentoring moved online for two summers with a focus on SARS-CoV-2 proteases (Lubin *et al.*, 2022 ▸, 2023 ▸; Burley *et al.*, 2020 ▸).

All RCSB PDB resources are accessible via https://www.rcsb.org/covid19, including the ∼3900 SARS-CoV-2 PDB structures currently available.

**Figure 2:**
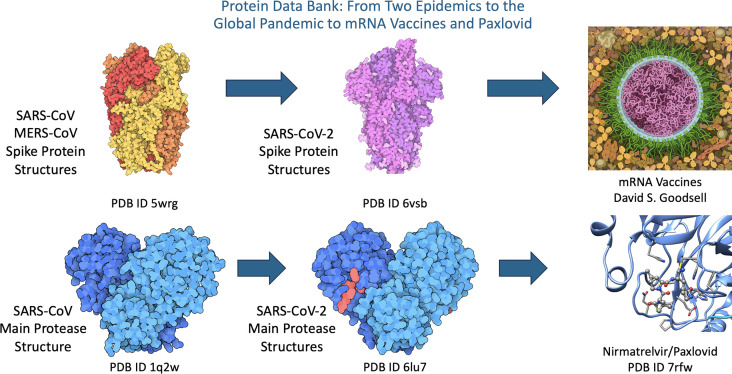
Coronavirus protein structures contributed to design of mRNA vaccines and facilitated structure-guided discovery of nirmatrelvir, the active ingredient of Pfizer’s Paxlovid anti-viral oral medication.

As a comprehensive data archive, the PDB contains structures of proteins from other coronaviruses, which provide important insights into viral pathogenesis. The 2003 outbreak of the closely related severe acute respiratory syndrome-related coronavirus (SARS) stimulated a steady flow of PDB structures for SARS and other coronaviruses. Many important scientific innovations that helped tame the COVID-19 pandemic were enabled and facilitated by decades of investment in structural biology and the PDB. Structural biologists and PDB data contributed to the design and rapid United States (US) Food and Drug Administration (FDA) Emergency Use authorization of two highly effective mRNA vaccines against SARS-CoV-2, and to the discovery, development and US FDA regulatory approval of Pfizer’s anti-viral Paxlovid (Fauci, 2022 ▸; Collins *et al.*, 2023 ▸), saving tens of millions of lives and preventing serious illness in hundreds of millions of infected individuals worldwide (Fig. 2 ▸).

## 2. The RCSB PDB website: enabling breakthroughs in research and education

The RCSB PDB research-focused web portal at RCSB.org not only provides users with free and open access to PDB data, but also offers a powerful suite of tools for searching, visualizing and analyzing these data. Additionally, every week RCSB PDB enriches the collection of structures with a set of annotations harvested from ∼50 trusted external resources [*e.g.* UniProt (UniProt Consortium, 2023 ▸), Comprehensive Antibiotic Resistance Database (Alcock *et al.*, 2020 ▸)] to provide biological, biochemical and evolutionary contexts for the structural information. These tools and data are accessed every day by researchers and their trainees, and educators and their students across different scientific fields and skill levels.

Users of RCSB.org can explore PDB data through either basic or advanced searches. Basic text searching is available from the box at the top of every RCSB.org page, in which users can search for structures by keywords, amino acid sequence or specific PDB ID(s). Alternatively, specialized search tools are offered from the advanced search menu: attribute search of specific data items for macromolecules and smaller chemical components, sequence similarity search, sequence motif search of small patterns, structure similarity search using BioZernike polynomials (Guzenko *et al.*, 2020 ▸), structure motif search of specific amino acids in specific 3D configurations (Bittrich *et al.*, 2020 ▸), and chemical similarity search to find bound ligands in the PDB. Advanced searches can combine multiple searches of specific types of data using Boolean logical operators to return data that comply with search criteria. For results containing multiple structures representing highly similar proteins, a grouping option generates a non-redundant search result set based on sequence identity or UniProt ID, and for similar structures deposited as a ‘Group’.

Individual structures can be explored through the *Structure Summary Pages* that provide high-level information about the entry, with additional tabs that offer a 3D structure view (Mol*), external structure annotations, experimental information, sequence annotations, genome alignments, ligand quality information and the versioning history of the data files.

Another specialized RCSB.org data delivery resource is the *Pairwise Structure Alignment* tool that calculates alignments using different trusted methods and displays sequence alignments and superposed 3D visualization. Comparisons can be made for any protein in the PDB archive and/or structures in uploaded data files, including CSMs.

### 2.1. Incorporation of computed structure models at RCSB.org

The field of structural biology has been transformed by the advent of robust software tools for protein structure prediction [*e.g.* *AlphaFold2* (Jumper *et al.*, 2021 ▸) and *RoseTTAFold* (Baek *et al.*, 2021 ▸)] for predicting monomeric and dimeric protein structures, with accuracy levels comparable to lower-resolution experimental methods. Importantly, these powerful artificial intelligence/machine learning (AI/ML)-based software tools for predicting protein structures from amino acid sequence information alone would not exist but for open access to the wealth of PDB data curated by the wwPDB (Burley & Berman, 2021 ▸). Structures predicted using these methods – referred to at RCSB.org as CSMs – in turn offer important value to researchers by serving as suitable alternatives and/or starting models for data analysis and hypothesis development when a desired experimental PDB structure is not available.

With the goal of providing a one-stop shop for studying 3D structures of biomolecules, RCSB.org provides parallel delivery to >1 million CSMs generated using *AlphaFold2* [from *AlphaFold DB* (Varadi *et al.*, 2022 ▸)] and *RoseTTAFold*/*AlphaFold2* (from *ModelArchive*, https://modelarchive.org/) alongside the collection of >210 000 experimental PDB structures. This two-pronged approach expands the number of structures available at RCSB.org by more than fivefold, providing users with access to structures covering the entire human proteome as well as those of model organisms, selected pathogens and organisms relevant to bioenergy research. Moreover, all CSMs are fully compatible with the same arsenal of RCSB PDB tools used to search, visualize and analyze experimental PDB data (Burley *et al.*, 2023 ▸), which are integrated weekly with related functional annotations from ∼50 trusted external resources, providing up-to-date information for each 3D biostructure. Interoperation of CSMs with all existing tools and features at RCSB.org was enabled by the extension of the PDBx/mmCIF data standard to establish the *ModelCIF* data standard for CSMs (Vallat *et al.*, 2023 ▸).

As RCSB.org supports a variety of users ranging in research interests and experience, a host of supporting information is provided in the form of website features, user experience design, documentation and training. The provenance of CSMs versus experimentally determined PDB structures is clearly and consistently identified throughout RCSB.org by a cyan-colored computer icon versus a dark-blue Erlenmeyer flask icon, respectively (Fig. 2 ▸). To prioritize use of experimentally determined PDB structures, CSMs are by default excluded from search results. Users are required to ‘opt-in’ to include CSMs using a toggle switch, to encourage their use only when experimental data are not available (Shao *et al.*, 2022 ▸; Moore *et al.*, 2022 ▸). When a user does ‘opt-in’ to include CSMs, prediction confidence is conveyed through the global and local (residue-level) model quality metrics [pLDDT, predicted local distance difference test (Tunyasuvunakool *et al.*, 2021 ▸)], which are presented on the search results page, structure summary pages, and through default coloring of the 3D structure images and visualizations (ranging from dark blue indicating regions of very high confidence to orange highlighting regions of very low confidence). In general, regions of lower prediction confidence (pLDDT < 70) should be ignored. To facilitate discovery of higher-quality CSMs, search results can be filtered to exclude CSMs of low prediction confidence based on the overall (or average) pLDDT value. Users are directed to the source CSM database (*e.g.* *AlphaFold DB*, *ModelArchive*) to download data [atomic coordinate data and predicted aligned error (PAE) files, when available]. The PAE datafile provided by *AlphaFold DB* provides prediction confidence estimates for inter-domain orientations.

Although the quality of structures produced by AI/ML methods is improving (Kryshtafovych *et al.*, 2023 ▸), there remains legitimate concerns regarding the trustworthiness of CSMs (Terwilliger *et al.*, 2024 ▸; Moore *et al.*, 2022 ▸). In particular, these methods face a number of limitations, such as in the prediction of ligand-binding sites and interactions, large-scale protein complexes and assemblies, and the existence of multiple conformational states that a macromolecule may adopt depending on its environment and neighboring interactions (Terwilliger *et al.*, 2024 ▸; Moore *et al.*, 2022 ▸). Summary pages for CSMs at RCSB.org contain a warning message (*‘There are no experimental data to verify the accuracy of this computed structure model. See Model Confidence metrics below for all regions of the polypeptide chain.’*) to encourage users to pay careful attention to CSM confidence metrics (*e.g.* pLDDT values, PAE information). Extensive documentation related to exploring CSMs at RCSB.org is available, with additional training materials and videos at PDB-101 (https://pdb101.rcsb.org/, see Section 3.2).

### 2.2. Data access via application programming interfaces

RCSB.org web services are powered by a set of application programming interfaces (APIs) that are freely available for users to access all search and data exploration tools programmatically (Bittrich *et al.*, 2023 ▸). The two primary APIs are a search API (https://search.rcsb.org), which supports the basic and advanced search services; and a data API (https://data.rcsb.org), which delivers all data and metadata associated with any given structure. Other APIs include the 1D coordinates API (https://1d-coordinates.rcsb.org) and the *ModelServer* API (https://models.rcsb.org/) for fetching sequence-level annotations and atomic coordinate data for a particular macromolecule of interest, respectively. Users can explore these APIs programmatically or through interactive query builder interfaces provided via RCSB.org. Additionally, a new Python client package for working with the search API service supports advanced searches through a Pythonic interface and syntax (see https://github.com/rcsb/py-rcsbsearchapi).

## 3. Looking ahead

Many challenges and opportunities are facing RCSB PDB, including the ever-growing number and complexity of experimental structures being deposited (particularly those coming from 3DEM), enhanced support for archiving integrative structures determined using data from complementary experimental methods, and the need to carefully archive structures that reveal microscopic details of chemical reactions in real time captured by serial crystallography using X-ray free-electron lasers and synchrotron radiation sources.

At the 2023 IUCr meeting, we highlighted ongoing efforts to develop the PDB-Dev prototype system for archiving integrative structures and the RCSB PDB training resources as two endeavors of particular interest to the community.

### 3.1. PDB-Dev prototype system supporting integrative or hybrid methods structural biology

Structural biologists are tackling ever larger and more complex macromolecular machines using integrative or hybrid methods (IHMs), which combine experimental measurements from complementary biophysical techniques. Integrative structure determination entails making measurements using complementary experimental methods (*e.g.* 3DEM and chemical cross-linking) and converting the results into spatial restraints that can be combined with known structures of component proteins and/or nucleic acids to determine structures of complex macromolecular assemblies. Anticipating this trend in 2015, a wwPDB IHM Task Force (https://www.wwpdb.org/task/hybrid) was assembled to make recommendations regarding data archiving and structure validation (Berman *et al.*, 2019 ▸; Sali *et al.*, 2015 ▸). As an interim measure, a standalone prototype system called PDB-Dev (https://pdb-dev.wwpdb.org/) was established for archiving integrative structures and making them publicly available (Vallat *et al.*, 2018 ▸, 2021 ▸; Burley *et al.*, 2017 ▸). PDB-Dev infrastructure supports data harvesting, deposition, validation, biocuration, archiving and dissemination of IHM biostructures that can span multiple spatiotemporal scales and conformational states. It is underpinned by the IHMCIF data standard (https://github.com/ihmwg/IHMCIF), another extension of the PDBx/mmCIF data standard (Westbrook *et al.*, 2022 ▸, 2005 ▸); a software library supporting the new data standard; a data harvesting system for collecting heterogeneous data from diverse experimental techniques; protocols for validating, biocurating and visualizing IHM structures; and web services for disseminating archived data. Like ModelCIF, IHMCIF enables interoperation with PDB data.

Work is currently underway to merge PDB-Dev structures, tools and workflows with the PDB. These IHM structures will complement existing PDB holdings and support basic and applied research focused on very large, conformationally dynamic biomolecular machines essential for survival of many living organisms and propagation of viruses.

Unification of PDB-Dev with PDB will support data collection and processing in parallel with the wwPDB OneDep system (Young *et al.*, 2017 ▸), and a parallel branch of the PDB archive will be established to house IHM structures.

At RCSB.org, features supported within the existing PDB-Dev web portal (Vallat *et al.*, 2021 ▸) will be made available to support IHM structure exploration alongside access to PDB experimental structures and CSMs, expanding our one-stop shop for studying 3D structures of biomolecules.

### 3.2. RCSB PDB training resources

The https://pdb101.rcsb.org/ web portal (hereafter PDB-101, meaning introductory) has provided training, outreach and education resources focused on structural biology since 2011 (Zardecki *et al.*, 2022 ▸). Training materials are provided with the goal of building confidence in current and future users in effectively utilizing RCSB.org tools and analyzing 3D biostructure data. ‘Molecule of the Month’ articles, now numbering nearly 290, introduce PDB data consumers to exciting new trends in structural biology and promote understanding of fundamental biology, biomedicine, bioenergy and biotechnology (Goodsell *et al.*, 2020 ▸ *b*; Goodsell *et al.*, 2015 ▸).

The PDB-101 *Guide to Understanding PDB Data* was created to help users navigate the contents of the PDB archive without the need of a detailed background in structural biology or data science. Topics cover biological assemblies, molecular graphics programs, *R* value and *R*_free_, and more. New articles are added as new structures are added to the archive and new capabilities are added to RCSB.org, such as ‘Exploring Carbohydrates in the PDB’ and ‘Computed Structure Models’.

Virtual training courses are intended to support graduate students, postdoctoral fellows and researchers covering data deposition through data exploration. Recordings and related materials are hosted at PDB-101. Announcements for new events are posted at RCSB.org and PDB-101; register for the training events newsletter at https://pdb101.rcsb.org/train/training-events.
